# Upregulation of the Long Noncoding RNA SNHG3 Promotes Lung Adenocarcinoma Proliferation

**DOI:** 10.1155/2018/5736716

**Published:** 2018-07-22

**Authors:** Liang Liu, Jianjiao Ni, Xinhong He

**Affiliations:** ^1^Department of Radiation Oncology, Fudan University Shanghai Cancer Center, Shanghai 200032, China; ^2^Department of Oncology, Shanghai Medical College, Fudan University, Shanghai 200032, China; ^3^Department of Interventional Radiology, Fudan University Shanghai Cancer Center, Shanghai 200032, China

## Abstract

Lung cancer is the leading cause of cancer-associated mortalities worldwide. Non-small-cell lung cancer (NSCLC) is the main reason for cancer-relevant death and constitutes 80% of lung cancer cases. Long noncoding RNAs (lncRNAs) have been found to be related to different kinds of cancer. Long noncoding RNAs played important roles in regulating the pathological and physiological processes of numerous cancers. To explore novel lung adenocarcinoma-associated lncRNAs, we analyzed the TCGA database and found that the lncRNA SNHG3 was significantly upregulated in lung adenocarcinoma. Bioinformatic analysis showed that SNHG3 may play key roles in regulating RNA splicing, tRNA processing, signal transduction, cell adhesion, transcription, and apoptosis. We also performed functional experiments to explore the roles of SNHG3 in lung adenocarcinoma cells. We found that SNHG3 promoted proliferation, cell cycle, and suppressed cell apoptosis of lung adenocarcinoma, suggesting that SNHG3 acted as an oncogene in lung adenocarcinoma. We believe that this study will provide a potential new therapeutic and prognostic target for lung adenocarcinoma.

## 1. Introduction

Lung cancer is the leading cause of cancer-associated mortalities worldwide [1, 2]. The prognosis of lung cancer is poor, with a 5-year survival rate of only 16% [3]. According to biological characteristics, lung cancer is classified into two types: non-small-cell lung cancer (NSCLC) and small-cell lung cancer (SCLC) [4]. NSCLC is the main reason for cancer-relevant death and constitutes 80% of lung cancer cases. Though many molecular genetic studies had been reported in lung adenocarcinoma, the precise molecular mechanisms underlying lung adenocarcinoma progression still remains to be further elaborated.

Long noncoding RNA (LncRNA) is a set of noncoding RNAs longer than 200 nucleotides and play important roles in regulating pathological and physiological processes of numerous cancers [5–9]. Abnormal expression of lncRNAs had been observed in different cancers, including prostate cancer [10], gastric cancer [6], breast cancer [11], and also lung cancer [12]. Recent reports had showed that many of the lncRNAs, including H19, HOTAIR, MALAT1, ANRIL, GAS5, and GAS5-AS1, were tumor-associated, especially in lung cancers [13–17]. For example, lncRNA GAS5-AS1 was significantly downregulated in NSCLC cells and inhibited NSCLC cell migration and invasion [18]. However, more novel NSCLC-associated lncRNAs still need to be further investigated.

More and more reports have provided evidence that the small nucleolar RNA host gene of lncRNA also acted as a key regulator in cancer progression. For example, SNHG1 promoted cell proliferation in non-small-cell lung cancer in You et al.'s report [19]. In this study, we aimed to identify and characterize differentially expressed lncRNAs in lung adenocarcinoma. TCGA analysis showed that SNHG3 was significantly upregulated in lung adenocarcinoma. A previous study on hepatocellular carcinoma showed that the expression of SNHG3 was overexpressed in cancer cells [20]. However, the molecular function of SNHG3 in lung adenocarcinoma remains unclear.

In our research, we focused on studying the molecular function of SNHG3 in lung adenocarcinoma. To explore the potential mechanisms of SNHG3 underlying lung adenocarcinoma progression, we performed GO and KEGG pathway analyses. We also overexpressed SNHG3 in lung adenocarcinoma cells and investigated its effect on the cell proliferation and apoptosis. We hoped that our study will provide a potential new therapeutic and prognostic target for lung adenocarcinoma.

## 2. Material and Method

### 2.1. Cell Culture

All cell lines were obtained from the American Type Culture Collection (Manassas, VA) which were confirmed by short tandem repeat (STR) analysis. All experiments were carried out with cell lines at passages below 30. The HEPES, nonessential amino acids, and sodium pyruvate were purchased from Gibco. A549 and NCI-H1299 cells were cultured in RPMI 1640 medium supplemented with 10% FBS (ExCell Bio, China) in a 37°C incubator with 5% CO_2_.

### 2.2. Plasmid Transient Transfection

The SNHG3 expression vector was generated by RT-PCR amplification of SNHG3 cDNA, using RNA from A549 cell lines. Full-length cDNA of SNHG3 was cloned into expression plasmid pcDNA3.1(+) (Invitrogen). Transfections were carried out with HilyMax (Dojindo Laboratories, Japan) following the manufacturer's protocol. After 48 h, overexpression of SNHG3 was confirmed by qRT-PCR.

### 2.3. Real-Time Quantitative Reverse Transcription PCR (qRT-PCR) Analysis

Total RNAs were extracted by using the TRIzol reagent (Sigma-Aldrich). Reverse transcript PCR was performed by using the PrimeScript RT reagent kit (Takara, Japan). Real-time quantitative PCR was carried out through AceQ qPCR SYBR Green Master Mix (Vazyme Biotech Co. Ltd.) on Roche LightCycler 480. Primers for SNHG3 were: forward, 5′-AGTGGTCGCTTCTTCTCCTTG-3′ and reverse, 5′-GATTGTCAAACCCTCCCTGTTA-3′. Primers for *β*-actin were: forward, 5′-CCTCTCCCAAGTCCACACAGTGACGCTGGGGCTGGCATTG-3′ and reverse, 5′-GGGCACGAAGGCTCATCATTGCTCTTGCTGGGGCTGGTGG-3′. Primers were synthesized by TransGen Biotech (Shanghai, China). The Ct values were normalized to estimate the different expression levels of genes with *β*-actin as reference. The expression level of SNHG3 was calculated by the 2^−ΔΔCt^ method. To ensure the quantitative accuracy, each sample was repeated 3 times.

### 2.4. Cell Proliferation Assay

We used Cell Counting Kit-8 (Dojindo Molecular Technologies Inc., Japan) to perform cell proliferation analysis in octuplicate according to the instructions of the manufacturer. A549 and H1299 cells of 3000 per well were seeded into 96-well plates, and examined at the time point of 0, 24, 48, and 72 h. At each time point, Cell Counting Kit-8 was added to the wells, and after an incubation of 2 h at 37°C, absorbance was measured at 450 nm with a Microplate Reader Expert 96 (Biochrom, UK).

### 2.5. Cell Cycle Assay

Cells were collected at 48 h after transfection. We used propidium iodide (PI) (Sigma-Aldrich, USA) to stain cells. After 15 min incubation in the dark, we used a FACStar flow cytometer (Becton-Dickinson, USA) to measure the cell cycle.

### 2.6. Annexin V-FITC Apoptosis Detection

Cells were harvested from the culture plates at 48 h after transfection. The Annexin V-FITC apoptosis detection kit (Sigma-Aldrich, USA) was used to measure apoptosis on a flow cytometer following the manufacturer's instructions.

### 2.7. GO and KEGG Analysis

GO analysis and KEGG analysis were applied to determine the biological roles of these differentially expressed mRNAs, based on the freely available online MAS system provided by the CapitalBio Corp. (Molecule Annotation System, http://bioinfo.capitalbio.com/mas3/). The *p* value (hypergeometric *p* value) denoted the significance of the pathway correlated to the conditions. The recommended *p* value cut off is 0.05.

### 2.8. PPI Network and Module Analysis

We used STRING online software to search the interaction relationships of the proteins encoded by DEGs, and the combined score > 0.4 was used as the cutoff criterion. We used Cytoscape software to visualize the PPI network.

### 2.9. Statistical Analysis

The numerical data were expressed as mean ± standard deviation (SD) of at least three determinations. According to the test condition, we used *t*-test or Mann–Whitney *U* test to perform statistical comparisons between groups of normalized data. A *p* < 0.05 was considered statistically significant with a 95% confidence level.

## 3. Results

### 3.1. LncRNA SNHG3 Was Upregulated in Lung Adenocarcinoma Tissue Samples

**Figure 1:**
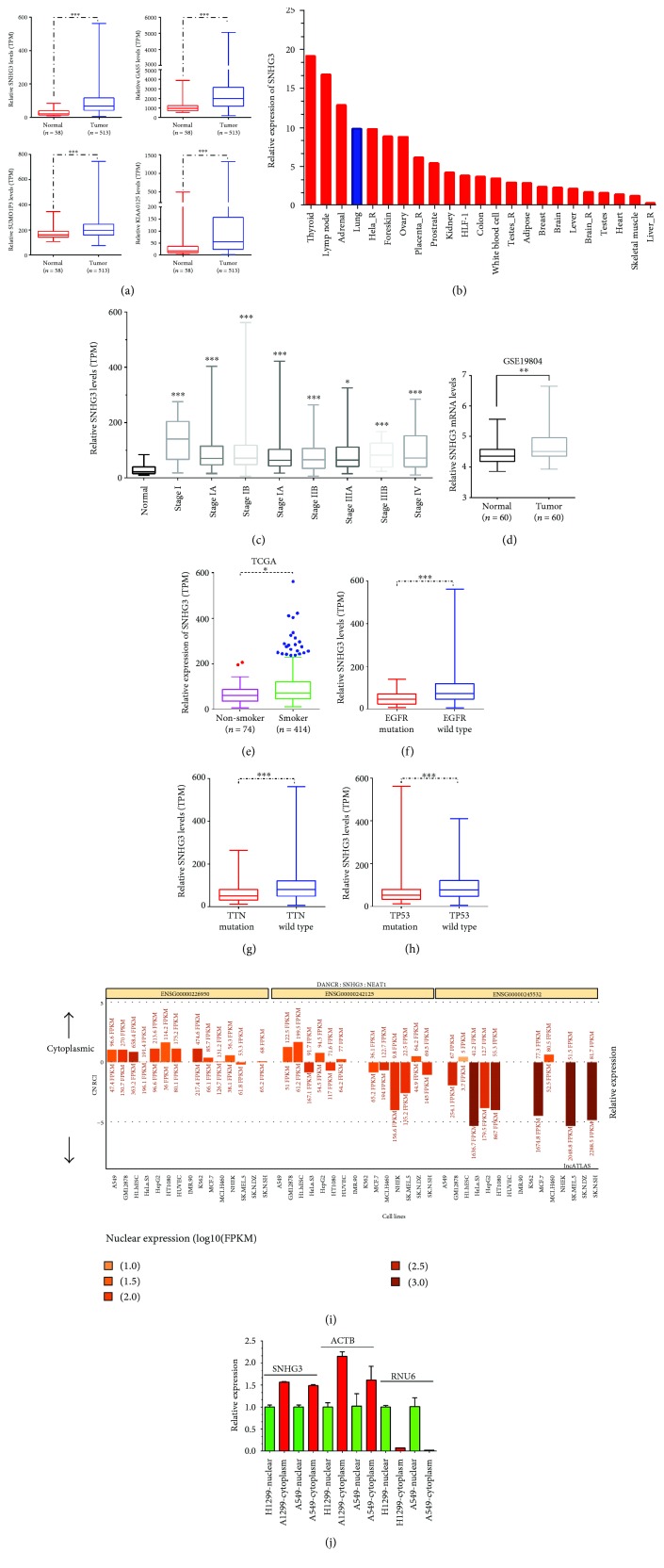
lncRNA SNHG3 was upregulated in lung adenocarcinoma. (a) SNHG3, GAS5, SUMO1P3, and KIAA0125 expression levels in lung adenocarcinoma tissues compared to normal lung tissues from the TCGA database. (b) The relative expression levels of SNHG3 in various human tissues from the NONCODE database. (c) SNHG3 was upregulated in all stages of lung adenocarcinoma compared to normal tissues. (d) SNHG3 was upregulated in lung adenocarcinoma compared to normal tissues by using GSE19804. (e–h) SNHG3 expression levels were correlated with the smoking status, EGFR mutation, TTN mutation, and TP53 mutation. (i) The lncATLAS dataset showed that SNHG3 was located in both the cytoplasm and nucleus. Cytoplasm-located DANCER and nucleus-located NEAT1 were used as positive controls (j). The cellular localization of SNHG3, ACTB, and RNU6 in NSLSC cell lines A549 and H1299. Significance was defined as *p* < 0.05 (^∗^*p* < 0.05, ^∗∗^*p* < 0.01, and ^∗∗∗^*p* < 0.001).

In this study, we analyzed the TCGA database to identify differentially expressed lncRNAs in lung adenocarcinoma. Our results showed that lncRNA SNHG3, GAS5, SUMO1P3, and KIAA0125 were significantly upregulated in tumors. Of note, SNHG3 showed the most significance between lung adenocarcinoma samples and adjacent normal tissues (Figure 1(a)). In the previous study, SNHG3 was found to be overexpressed in hepatocellular carcinoma, showing that SNHG3 acted as an oncogene. NONCODE data showed that SNHG3 expression was significantly higher in the thyroid gland, lymph node, adrenal gland, and lungs, suggesting that SNHG3 may play a key role in lung cancer progression (Figure 1(b)). To evaluate the possible prognostic value of SNHG3, we analyzed the correlation between expression levels of SNHG3 and pathological parameters in TCGA. We found that SNHG3 was upregulated in all stages compared to normal tissues (Figure 1(c)). Moreover, we analyzed the other public dataset, GSE19804, which included 60 normal lung samples and 60 non-small-cell lung cancer samples. GSE19804 data analysis showed similar results with the TCGA data analysis where SNHG3 was upregulated in NSCLC samples compared to normal samples (Figure 1(d)).

Interestingly, upregulation of SNHG3 was also correlated with the smoking status (Figure 1(e)). Furthermore, we found that SNHG3 was significantly downregulated in NSCLC with EGFR mutation, TTN mutation, and TP53 mutation compared to wild type NSCLC (Figures 1(f)–1(h)).

To investigate the specific roles of SNHG3 in lung adenocarcinoma cells, we also detected the cellular localization of SNHG3 in A549 and H1299 cells by separating cells into the nucleus and cytoplasmic fractions. RNA fractionation analyses revealed that SNHG3 was located in both the nucleus and cytoplasm, while we detected the cellular localization of ACTB and RNU6 in A549 and H1299 cells and found that ACTB was mainly located in the cytoplasm and RNU6 was mainly located in the nucleus (Figure 1(j)). These results showed that the cellular localization of SNHG3 was similar to ACTB. Moreover, we analyzed the lncATLAS dataset to reveal the cellular localization of SNHG3 in a series of cell lines, including A549 (Figure 1(i)). This analysis also suggested that SNHG3 was located both in the nucleus and cytoplasm.

### 3.2. Upregulation of SNHG3 Predicted a Poor Prognosis in Lung Cancer

**Figure 2:**
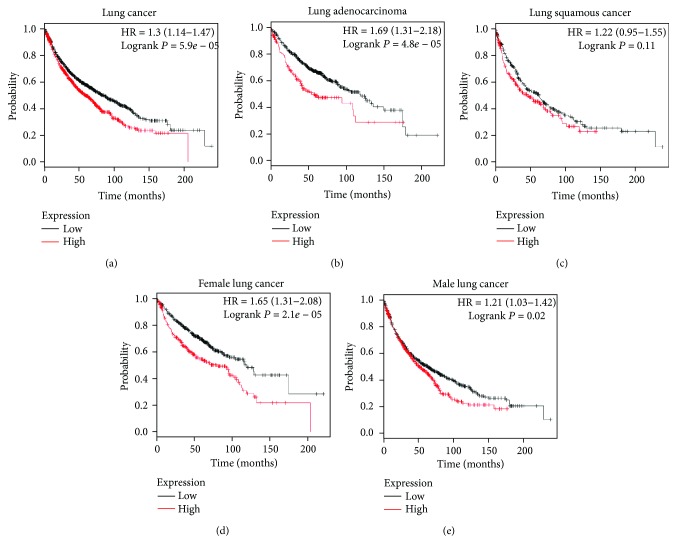
Upregulation of SNHG3 predicted a poor prognosis in lung cancer. The overall survival time in the SNHG3-high group was lower than that in the SNHG3-low group in lung cancer (a), lung adenocarcinoma (b), female lung cancer (d), and male lung cancer (e), but not in lung squamous cancer (c).

Furthermore, the Kaplan-Meier plotter database was used to comprehensively analyze the association of lncRNA SNHG3 expression with the survival rates in various types of lung cancer. As shown in Figure 2, we found that the overall survival time in the SNHG3-high group was lower than that in the SNHG3-low group in lung cancer, lung adenocarcinoma, female lung cancer, and male lung cancer. However, we also observed that the expression levels of SNHG3 was not associated with the overall survival time in lung squamous cancer.

### 3.3. Molecular Function Analysis of SNHG3

**Figure 3:**
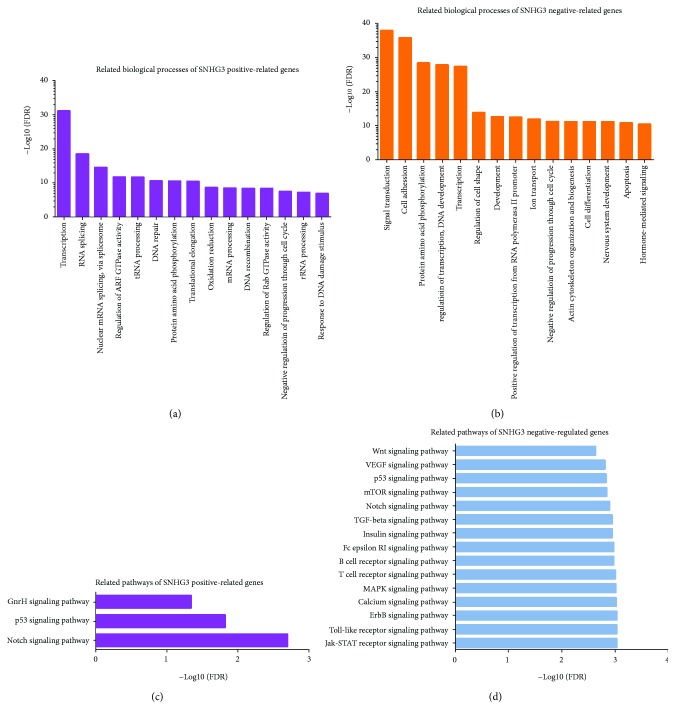
GO and KEGG pathway analyses of SNHG3. GO (a and b) and KEGG (c and d) pathway analyses of the top 500 differentially expressed genes using MAS 3.0. (a) GO biological process analysis of SNHG3-positive genes and SNHG3-negative genes (b). (c) KEGG pathway enrichment analysis of SNHG3-positive genes and SNHG3-negative genes (d) using MAS 3.0

To predict the functions of SNHG3 in lung adenocarcinoma, we constructed a gene coexpression network according to Pearson correlation coefficients using the TCGA database. Next, the top 500 positive-related genes and the top 500 negative-related genes were classified according to the GO term using MAS 3.0. According to our analysis, we found that SNHG3 positive-related genes were associated with transcription, RNA splicing, nuclear mRNA splicing, regulation of ARF GTPase activity, and tRNA processing. SNHG3 negative-related genes were mainly involved in regulating signal transduction, cell adhesion, transcription, and apoptosis (Figures 3(a) and 3(b)). KEGG pathway analysis suggested that positive-related genes of SNHG3 were mainly enriched in Notch signaling pathway, p53 signaling pathway, and GnRH signaling pathway, and negative-related genes of SNHG3 were mainly enriched in Jak-STAT signaling pathway, Toll-like receptor signaling pathway, MAPK signaling pathway, calcium signaling pathway, Wnt signaling pathway, and ErbB signaling pathway (Figures 3(c) and 3(d)).

**Figure 4:**
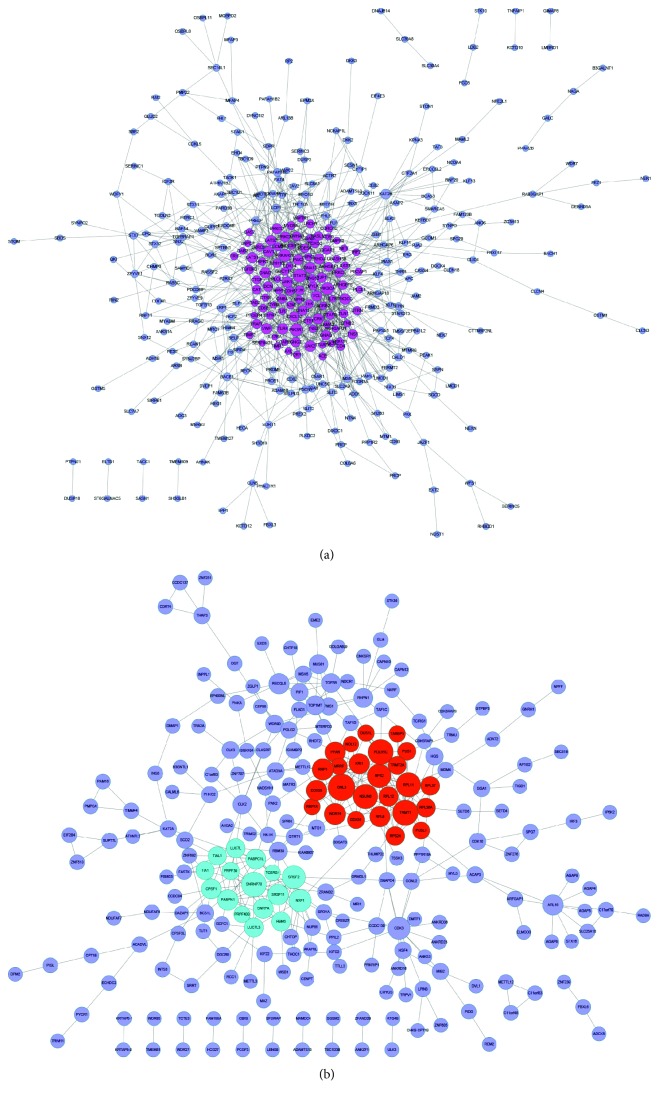
(a) The gene coexpression networks of SNHG3. (b) The PPI network of SNHG3.

To evaluate the interactive relationships among SNHG3 coexpressed genes, we mapped the DEGs to STRING, and only experimentally validated interactions with a combined score > 0.4 were selected as significant. Then, we used Cytoscape software to construct PPI networks in Figure 4.

### 3.4. Construction of SNHG3-Regulated lncRNA-Mediated ceRNA Networks in Lung Adenocarcinoma

**Figure 5:**
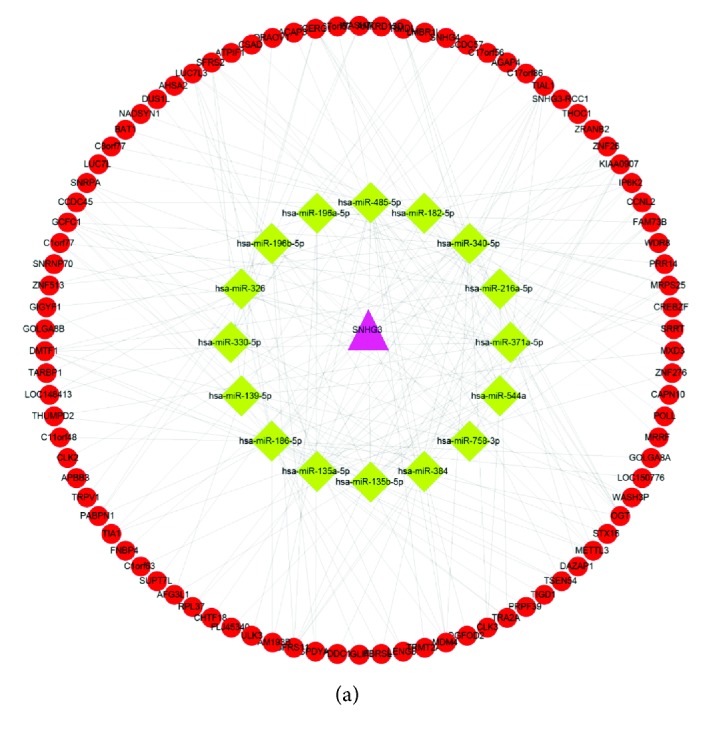
(a) SNHG3 regulated lncRNA-mediated ceRNA networks. The networks contain 16 miRNAs and 90 mRNAs.

To identify SNHG3-mediated ceRNA networks, we first screened SNHG3-binding miRNAs by using the StarBase database. Next, we explored target mRNAs of miRNA by using StarBase and Targetscan. Finally, a coexpression network based on the correlation analysis between the differentially expressed mRNAs and lncRNAs was constructed (Figure 5(a)). The top 500 positive SNHG3-mRNA interactions were integrated into the coexpression networks according to the positive regulation. The results showed that SNHG3-mediated ceRNA networks contain 16 miRNAs and 90 mRNAs. The networks were drawn using Cytoscape 3.0.

### 3.5. Overexpression of SNHG3 Promoted Lung Adenocarcinoma Cell Growth in Vitro

**Figure 6:**
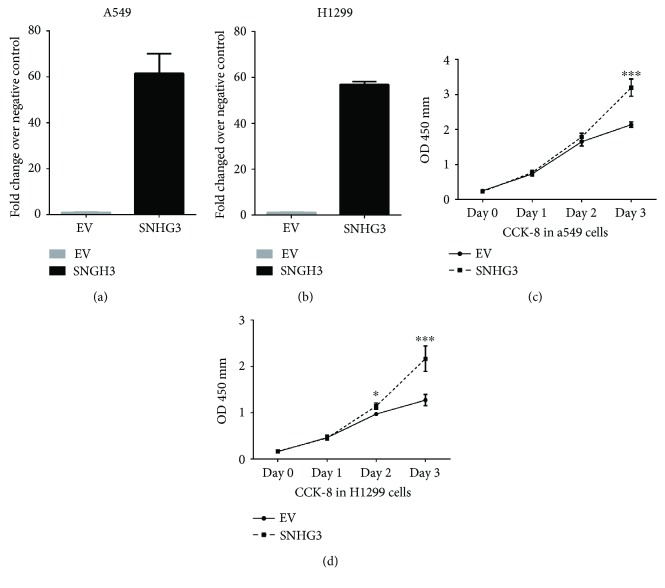
SNHG3 promotes cell proliferation in NSLSC cell lines A549 and H1299. (a and b) The overexpression of SNHG3 in NSLSC cell lines A549 and H1299 was measured by RT-PCR. (c and d) Cell proliferation analysis was performed with the CCK-8 assay in A549 and H1299 cells. Cells transfected with SNHG3 were seeded into a 96-well plate at 5000 cells/well. Significance was defined as *p* < 0.05 (^∗^*p* < 0.05 and ^∗∗∗^*p* < 0.001).

Aiming to evaluate the functions of SNHG3 in lung adenocarcinoma, we transfected A549 and H1299 cells with the expression plasmid pcDNA 3.1(+)-SNHG3. We observed a significant increase of SNHG3 gene expression in the transfected cells (Figures 6(a) and 6(b)).

To characterize the role of SNHG3 in regulating cell proliferation in lung adenocarcinoma cell lines, we performed a CCK-8 assay in A549 and H1299 cells. As shown in Figures 6(c) and 6(d), overexpression of SNHG3 significantly promoted proliferation of A549 and H1299 cells.

### 3.6. Overexpression of SNHG3 Promoted Lung Adenocarcinoma Cell Cycle Progression

**Figure 7:**
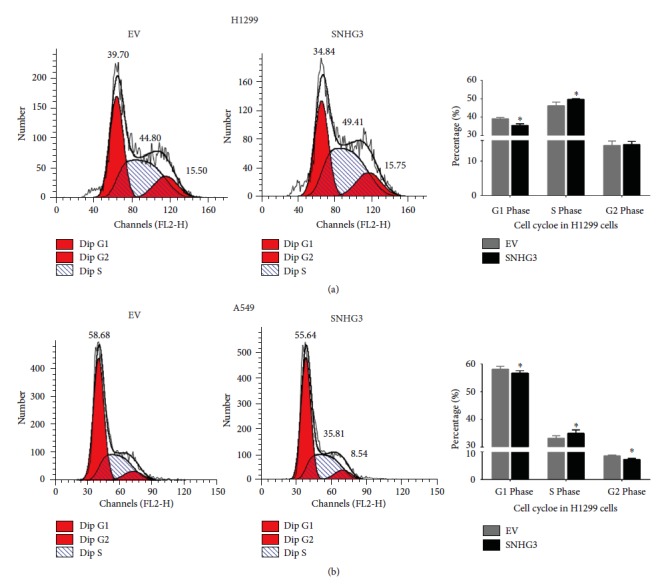
SNHG3 promotes cell cycle in NSLSC cell lines A549 and H1299. Cell cycle assay was performed in H1299 and A549 cells. Cells were transfected with SNHG3 for 48 h, stained with PI, and evaluated with a FACScalibur flow cytometer. Overexpression of SNHG3 decreased the percentage of cells in the G1 phase and increased the percentage of cells in the S phase compared to the negative control. Significance was defined as *p* < 0.05 (^∗^*p* < 0.05).

In this study, we also measured the function of SNHG3 on the cell cycle profile in A549 and H1299 cells. We found that overexpression of SNHG3 in A549 and H1299 cells decreased the percentage of cells in the G1 phase and increased the percentage of cells in the S phase (Figures 7(a) and 7(b)). Taken together, these data indicated that SNHG3 increased the proliferation and growth of A549 and H1299 cells by promoting cell cycle progression.

### 3.7. Overexpression of SNHG3 Inhibited Lung Adenocarcinoma Cell Apoptosis

**Figure 8:**
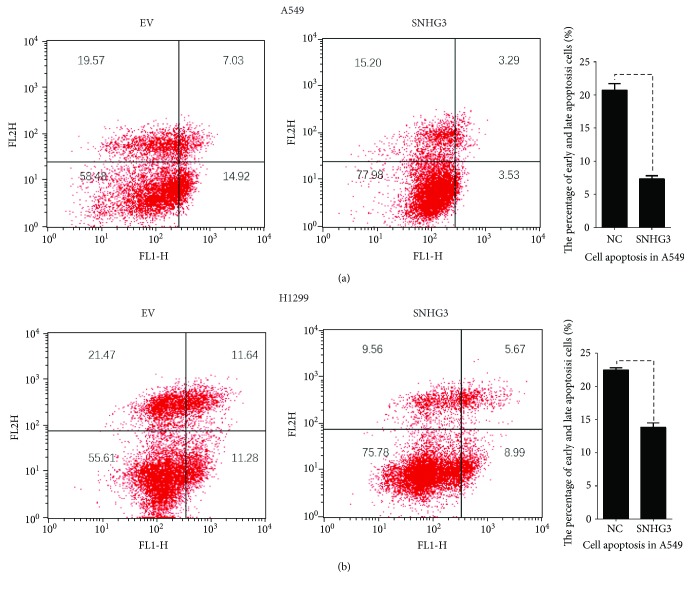
SNHG3 inhibited cell apoptosis in NSLSC cell lines A549 and H1299. Cell apoptosis assay was performed with a flow cytometer. Cells were transfected with SNHG3 for 48 h, and subjected to a cell apoptosis assay. Overexpression of SNHG3 in A549 and H1299 cells decreased the fraction of both early apoptotic cells and late apoptotic cells.

Then, we studied the role of SNHG3 in apoptosis in lung adenocarcinoma. We found that upregulation of SNHG3 in A549 and H1299 cells inhibited early and late apoptosis in lung adenocarcinoma (Figures 8(a) and 8(b)).

## 4. Discussion

Long noncoding RNA played important roles in regulating pathological and physiological processes of numerous cancers, including lung adenocarcinoma. In this study, we demonstrated that SNHG3 was upregulated in lung adenocarcinoma. GO and KEGG pathway analyses showed that SNHG3 may play key roles in regulating signal transduction, cell adhesion, protein amino acid phosphorylation development, and apoptosis. A SNHG3-mediated competing endogenous RNA network was also constructed. To further explore the molecular function of SNHG3 in lung adenocarcinoma, we overexpressed SNHG3 and found that SNHG3 promoted lung adenocarcinoma cell proliferation, cell cycle, and suppressed cell apoptosis.

LncRNA was reported to play important roles in regulating the pathological and physiological processes of numerous cancers. Increasing reports showed that many lncRNAs including RMRP, PVT1, HOTAIR, HIT, and TUG1 showed disease-associated dysregulation in lung cancer [12, 21–24]. Here, we observed that SNHG3 was significantly upregulated in lung adenocarcinoma. Moreover, we found that upregulation of SNHG3 was significantly correlated with smoking status, EGFR mutation, TP53 mutation, and TTN mutation. We also found the overall survival time in the SNHG3-high group was lower than that in the SNHG3-low group in lung cancer, NSCLC, female lung cancer, and male lung cancer.

In lung cancer, only a small part of lncRNAs, such as H19, HOTAIR, MALAT1, ANRIL, and GAS5 have been identified to be tumor-associated especially in lung cancers. For example, lncRNA GAS5-AS1 was significantly downregulated in NSCLC cells and inhibited NSCLC cell migration and invasion. However, more novel lung adenocarcinoma-associated lncRNAs still need to be further investigated. In this study, we performed GO and KEGG pathway analyses to explore the SNHG3 molecular function based on coexpression analysis. We found that SNHG3 positive-related genes were associated with RNA splicing and tRNA processing. SNHG3 negative-related genes were mainly involved in regulating signal transduction, cell adhesion, transcription, and apoptosis. Moreover, we constructed a SNHG3-mediated competing endogenous RNA network to explore the molecular mechanisms involved in SNHG3 regulating lung adenocarcinoma progression.

A previous study in hepatocellular carcinoma showed that the expression of SNHG3 was overexpressed in cancer cells. However, the molecular function of SNHG3 in lung adenocarcinoma remains unclear. To explore the function of SNHG3 on lung adenocarcinoma, we first detected the cellular localization of SNHG3 in A549 and H1299 cells. RNA fractionation analyses revealed that SNHG3 was both in the nucleus and cytoplasm. We also overexpressed SNHG3 in lung adenocarcinoma cells and found that enhanced SNHG3 expression could significantly promote lung adenocarcinoma cell proliferation, cell cycle, and suppressed cell apoptosis. Taken together, these results suggested that SNHG3 acted as an oncogene in lung adenocarcinoma.

Several limitations of this study should be noted. First, the expression levels of SNHG3 in lung adenocarcinoma samples should be detected to confirm the significance of the signature. Second, additional molecular investigations of SNHG3 in lung cancer was still needed. For example, whether and how SNHG3 functioned as a ceRNA should be further explored.

In conclusion, we demonstrated that SNHG3 expression was upregulated in lung adenocarcinoma and overexpression of SNHG3 promoted SNHG3 cell proliferation. Therefore, SNHG3 might serve as a therapeutic target in lung adenocarcinoma.
